# Aryl Hydrocarbon Receptor Signaling Is Functional in Immune Cells of Rainbow Trout (*Oncorhynchus mykiss*)

**DOI:** 10.3390/ijms21176323

**Published:** 2020-08-31

**Authors:** Jun-Young Song, Ayako Casanova-Nakayama, Anja-Maria Möller, Shin-Ichi Kitamura, Kei Nakayama, Helmut Segner

**Affiliations:** 1 Centre for Fish and Wildlife Health, Department of Infectious Diseases and Pathobiology, Vetsuisse Faculty, University of Bern, 3012 Bern, Switzerland; jysong2012@korea.kr (J.-Y.S.); ayako0109@yahoo.com (A.C.-N.); anjamamoe@gmail.com (A.-M.M.); 2 Center for Marine Environmental Studies (CMES), Ehime University, Matsuyama, Ehime 790-8577, Japan; kitamura.shinichi.mx@ehime-u.ac.jp (S.-I.K.); nakayama.kei.mj@ehime-u.ac.jp (K.N.)

**Keywords:** aryl hydrocarbon receptor, immune cells, head kidney, fish, rainbow trout, immunotoxicity, cytochrome P4501A, immune signaling

## Abstract

The arylhydrocarbon receptor (AhR) is an important signaling pathway in the immune system of mammals. In addition to its physiological functions, the receptor mediates the immunotoxic actions of a diverse range of environmental contaminants that bind to and activate the AhR, including planar halogenated aromatic hydrocarbons (PHAHs or dioxin-like compounds) and polynuclear aromatic hydrocarbons (PAHs). AhR-binding xenobiotics are immunotoxic not only to mammals but to teleost fish as well. To date, however, it is unknown if the AhR pathway is active in the immune system of fish and thus may act as molecular initiating event in the immunotoxicity of AhR-binding xenobiotics to fish. The present study aims to examine the presence of functional AhR signaling in immune cells of rainbow trout (*Oncorhynchus mykiss*). Focus is given to the toxicologically relevant AhR2 clade. By means of RT-qPCR and in situ hybdridization, we show that immune cells of rainbow trout express *ahr 2α* and *ahr 2β* mRNA; this applies for immune cells isolated from the head kidney and from the peripheral blood. Furthermore, we show that in vivo as well as in vitro exposure to the AhR ligand, benzo(a)pyrene (BaP), causes upregulation of the AhR-regulated gene, *cytochrome p4501a*, in rainbow trout immune cells, and that this induction is inhibited by co-treatment with an AhR antagonist. Taken together, these findings provide evidence that functional AhR signaling exists in the immune cells of the teleost species, rainbow trout.

## 1. Introduction

The arylhydrocarbon receptor (AhR) is a cytosolic protein which belongs to the basic helix-loop-helix (bHLH)-PAS family (*Per* (period)-*ARNT* (AhR nuclear translocator)-*Sim* (single-minded)) [1,2,3]. Canonical AhR signaling involves the ligand-dependent activation of the receptor, followed by translocation into the nucleus where it forms a dimer with ARNT. This receptor complex then interacts with specific DNA binding sites, termed AHR response elements (AHREs), on the target genes [3,4]. While the nature of the endogenous, physiological ligands of the AhR is still under debate [5,6], it is clear that a range of environmental contaminants including planar halogenated aromatic hydrocarbons (PHAHs or dioxin-like compounds) and polynuclear aromatic hydrocarbons (PAHs) are high affinity ligands of the AhR [7,8]. Activation of the AhR pathway by environmental contaminants induces a battery of genes involved in the biotransformation of xenobiotics, and it can lead to diverse toxic responses including developmental abnormalities, cancer promotion, liver and renal failure, and immunotoxicity [9,10,11,12]. It was this role in mediating the toxicity of dioxins and related compounds why the AhR has attracted much attention in toxicological research, and in fact, it was the observation that dioxins can induce biotransformation enzymes like cytochrome P450 monooxygenases which led to the discovery of the AhR [3,13,14].

The AhR is present in all vertebrate classes although there exist differences with respect to AhR diversification: while mammals possess a single AhR gene, fishes have multiple AhR genes [15,16]. For most bony fish species studied so far, two AhR clades have been identified, AhR1 and AhR2, which arose by gene duplication occurring early in vertebrate evolution [17,18]. The piscine AhR1 is orthologous to the mammalian AhR, whereas the AhR2 clade is a novel form in teleost fish [19,20,21]. 

In mammals, immunotoxicity is a prominent toxic effect resulting from exposure to AhR-binding contaminants [22,23,24,25,26]. The immunotoxic activity of AhR ligands is related to the fact that the AhR plays an important physiological role in the mammalian immune system [2,6,8,27,28,29]. The receptor is involved, among others, in the proliferation and differentiation of immune cells, and in the regulation of inflammatory responses, particularly in barrier tissues like skin and gut [8,12]. Dysregulation of AhR signaling by environmental receptor ligands can disturb, for instance, the balance between Th17 and other T cell subsets, the formation of intestinal lymphoid follicles, or B cell differentiation and antibody secretion. The disruption of immune functions by AhR-binding xenobiotics may eventually lead to immunotoxicity [2,8,12,30]. 

For fishes, it is well established that exposure to AhR-binding PAHs and PHAHs is associated with disturbed immune functions and increased disease susceptibility. This has been shown both in field and in laboratory studies [31,32,33,34,35,36,37,38,39,40,41,42]. The highly vascularized immune organs and the circulating immune cells are directly exposed to absorbed contaminants and in fact they accumulate AhR-binding chemicals [43,44]. These facts point to the possibility that the known immunotoxic effects of PHAHs and PAHs to fish are mediated via AhR signaling in the piscine immune system. Surprisingly, however, the question upon the existence of a functional AhR pathway in the teleostean immune cells has hardly been studied to date. 

The present study aims to examine the presence of functional AhR signaling in immune cells of rainbow trout (*Oncorhynchus mykiss*). We focus on the AhR2 clade, since the receptors of this clade mediate the effects of PHAHs and PAHs in fish [18,19,45,46]. The AhR2 isoforms were shown to play a key role in the developmental and cardiovascular toxicity of dioxin-like chemicals in several fish species including rainbow trout, and they contribute to the developmental toxicity of PAHs [45,47,48]. Beyond, there exists evidence that AhR2 signaling is involved in the toxic effects of HAHs and PAHs on the reproductive system of fish [11,49]. In rainbow trout, two AhR2 isoforms are present, AhR2α and AhR2β, which share 98% amino acid identity but differ in their functional properties [50]. Using RT-qPCR and in situ hybridization, we examine the presence of *ahr2α* and *ahr2β* mRNA in mature immune cells from the peripheral circulation as well as in developing immune cells from the head kidney, a main hematopoietic organ of trout. Both control fish and fish exposed to the AhR ligand, benzo(a)pyrene (BaP) [51], are investigated. In order to obtain insight if the AhR is functional in the immune cells, we evaluate whether exposure of trout to BaP induces an upregulation of the AhR-regulated gene, *cytochrome P4501A* (*cyp1a*), and whether this can be inhibited by the AhR antagonist, α-naphthoflavone. In previous studies, our group could show that rainbow trout immune cells possess inducible CYP1A protein [52], and that BaP exposure of trout leads to an altered composition of the peripheral immune cells, particularly of the T cells [53]. Both findings provide indirect evidence for the presence of a functional AhR pathway in the immune system of rainbow trout. Here we now aim to directly demonstrate the existence of AhR signaling in trout immune cells. 

## 2. Results

### 2.1. Fluorescent In Situ Hybridization (FISH) of AhR2

**Figure 1:**
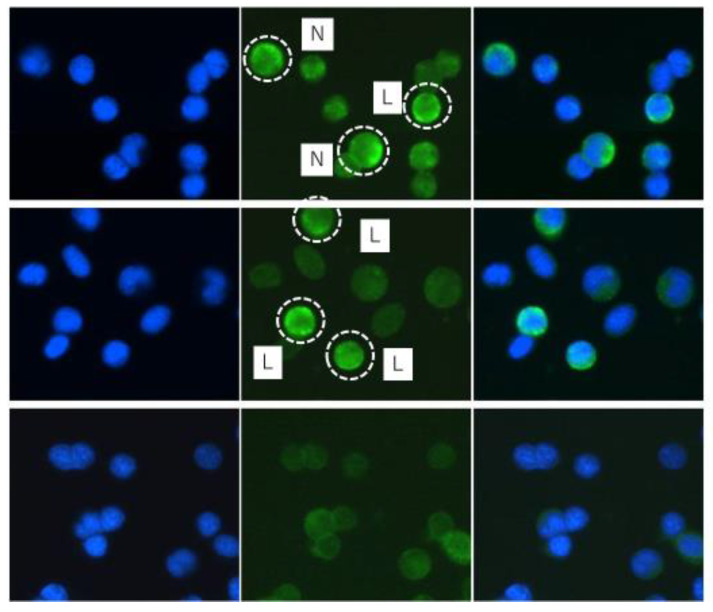
Fluorescent In Situ Hybridization (FISH) with the AhR probe (AhR2α + β) on the immune cells isolated from peripheral blood. Left: fluorescent nuclear staining with DAPI (blue), middle: fluorescent AhR mRNA staining (green), right: merge of nuclear and AhR-RNA staining. The first and second line show cells hybridized with the AhR-antisense probe (positive probe), the third line shows cells stained with the AhR sense probe (negative probe). The circles indicate positive immune cells: L = lymphocyte-like morphology; N = larger polymorphonuclear cells ressembling neutrophils.

For FISH we developed an antisense probe detecting *ahr2*(*α* + *β*) mRNA. Using this probe, we could demonstrate the presence of *ahr2*(*α + β*) mRNA in immune cells isolated from the peripheral blood of rainbow trout (Figure 1). The *ahr2*(*α* + *β*)-positive cells included immune cells with a small cytoplasmic rim around the nucleus as well as larger cells with segmented, polymorphic nuclei and a broader cytoplasmic rim. Importantly, only a sub-fraction but not all immune cells stained positive for *ahr2*(*α* + *β*) mRNA. The *ahr2*(*α* + *β*) mRNA could be detected in immune cells of both control and BaP-exposed rainbow trout. 

### 2.2. Cyp1a Gene Expression

**Figure 2:**
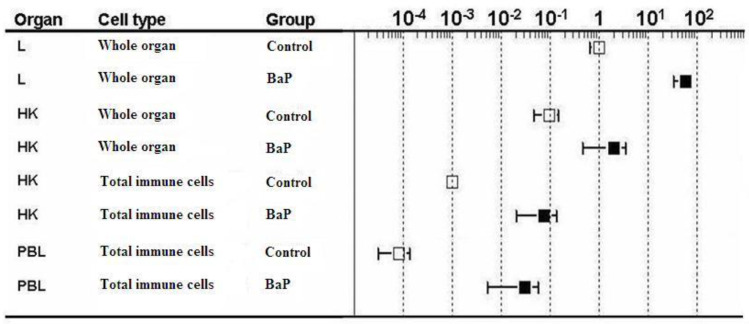
*cyp1a* gene expression levels of liver (L), head kidney (HK), immune cells isolated from head kidney and immune cells isolated from peripheral blood (PBL). The immune cells were not sorted but the total population was used. The RT-qPCR data were calculated using the 2^−(∆∆ct)^ method. Gene expression levels of the liver of control rainbow trout were set to be 1 and the mRNA expression levels of the other treatments were expressed relative to the control liver (note: log scale on the top *x*-axis). Data represent mean values ± SD from 10 fish per treatment. PBL = peripheral blood immune cells.

In control trout, *cyp1a* transcript levels were higher in the liver than in the head kidney and in the immune cells isolated from either head kidney or blood (Figure 2, white boxes). The head kidney displayed significantly higher *cyp1a* mRNA levels than the isolated immune cells of head kidney and blood. The lowest *cyp1a* expression was found in the immune cells from the peripheral blood. When the rainbow trout were exposed to BaP, *cyp1a* transcript levels were elevated in the organs and cells, but the relative ranking of cyp1a mRNA expression remained unchanged (liver > head kidney > head kidney immune cells > blood immune cells) (Figure 2, black boxes). The BaP-induced increase of *cyp1a* transcript levels relative to controls was highest in the isolated blood immune cells (92 times higher expression than in blood immune cells of control trout, Table 1). 

**Figure 3:**
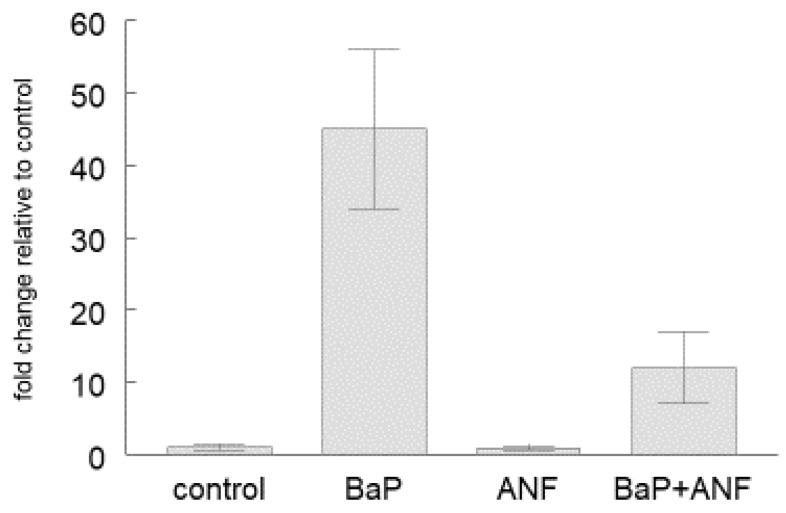
Inhibition of BaP induction of *cypa1* mRNA by ANF. Cultured head kidney immune cells were incubated for 48 h either under control conditions, under exposure to 0.5 μM benzo(a)pyrene (BaP), under exposure to 1 μM α-naphtoflavone (ANF), or a combination of 0.5 μM + 1 μM ANF. Cells were sampled after 48 hrs, extracted and analysed for *cyp1a* mRNA by mean of RT-qPCR. Data are mean values of three independent in vitro incubations. The *cyp1a* mRNA levels in trout immune cells exposed to (BaP + ANF) are significantly (*p* < 0.05) lower than those in trout immune cells exposed to BaP.

In order to test if the CYP1A induction is mediated through the AhR pathway, we exposed cultured head kidney immune cells to BaP or to BaP in combination with the AhR antagonist, ANF (BaP + ANF). Presence of ANF significantly reduced the BaP-dependent upregulation of *cyp1a* mRNA (Figure 3).

### 2.3. ahR2α Gene Expression

**Figure 4:**
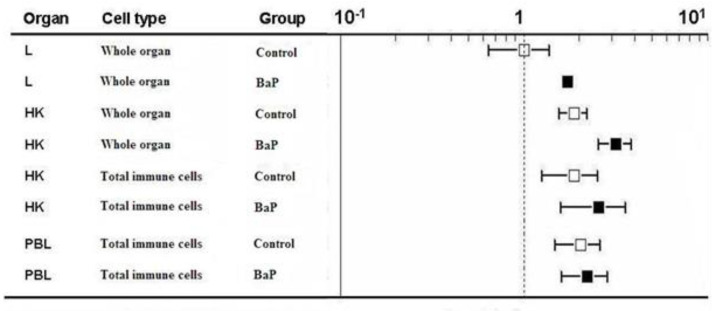
*ahr2α* gene expression levels of liver (L), head kidney (HK), immune cells isolated from head kidney and immune cells isolated from peripheral blood (PBL) are shown. The immune cells were not sorted but the total population was used. The RT-qPCR data were calculated using the 2^−(∆∆ct)^ method. Gene expression levels of the liver of control rainbow trout were set to be 1 and the mRNA expression levels of the other treatments were compared to the control liver (note: log scale on the top *x*-axis). Data represent mean values ± SD from 10 fish per treatment. PBL = peripheral blood immune cells.

Transcription of the *ahr2α* gene tended to be slightly higher in the immune organs and cells of control trout compared to the liver of control trout (Figure 4, white boxes). In contrast to *cyp1a* mRNA, there was no clear difference of *ahr2α* transcript levels between the head kidney organ and the immune cells. The BaP treatment led to a slight but significant upregulation of *ahr2α* mRNA in the liver and in the head kidney, but not in the immune cells (Figure 4, black boxes, Table 2). 

### 2.4. ahr2β Gene Expression

**Figure 5:**
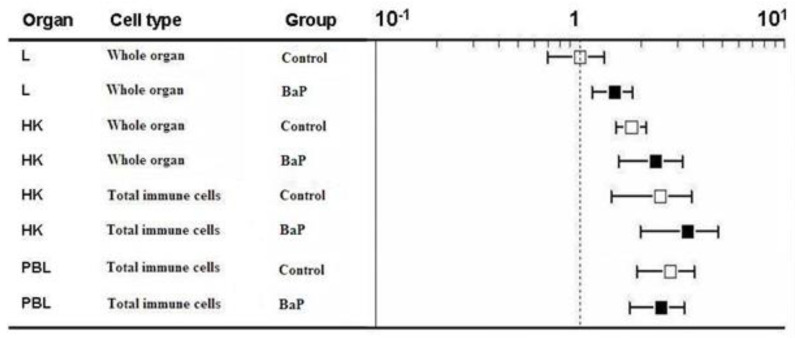
*ahr2β* gene expression levels of liver (L), head kidney (HK), immune cells isolated from head kidney and immune cells from peripheral blood (PBL) are shown. The immune cells were not sorted but the total population as obtained from the isolation process was used. The RT-qPCR data were calculated using the 2^−(∆∆ct)^ method. Gene expression level of the liver of control rainbow trout was set to be 1 and the mRNA expression levels of the other treatments were compared to the control liver (note: log scale on the top *x*-axis). Data represent mean values ± SD from 10 fish per treatment. PBL = peripheral blood immune cells.

As already observed for *ahr2α*, the transcription of *ahr2β* tended to be higher in the head kidney and the immune cells compared to the liver (Figure 5), although the differences were not significant. The tendency for higher *ahr2β* expression in the immune organs and cells was present in both control and BaP-eposed fish. There was no significant difference of *ahr2β* mRNA expression between the head kidney and the immune cells isolated from the head kidney. Exposure to BaP increased the expression of *ahr2β*, with the exception of the blood immune cells (Table 3). The BaP-induced upregulation was not significant, neither in the liver, nor in the head kidney and immune cells. 

## 3. Discussion

Environmental contaminants such as planar dioxins, PCBs and PAHs that bind to and activate the AhR are known to impair immune function and to increase pathogen susceptibility of teleost fish. Evidence for this comes from laboratory experiments as well as from field studies [31,32,33,35,36,39,40,42,54,55]. In mammals, it is well established that the immunotoxic actions of PHAHs and PAHs are related to their interference with the AhR signaling pathway in the immune system (e.g. [12,25,56,57]). This leads to the question whether the AhR pathway may also be involved in the immunotoxicity of PHAHs and PAHs to fish. The first step to answer this question is to demonstrate the presence of functional AhR signaling in the immune cells of fish. To this end, the present study examined if immune cells of rainbow trout express transcripts of the *ahr2* clade, which mediates dioxin toxicity in teleosts, and if BaP exposure results in the induction of the AhR-regulated *cyp1a* gene in the immune organs and cells. The induction of *cyp1a* mRNA and its inhibition by the AhR antagonist, ANF was taken as proxy to indicate functional AhR signaling. 

By means of RT-qPCR we could demonstrate the presence of *cyp1a* transcripts in the head kidney and in the immune cells of rainbow trout. The observation of *cyp1a* mRNA in the head kidney organ agrees with the results of previous reports that showed the presence of cyp1a protein and cyp1a enzyme activity in haematopoietic organs of fish [44,58,59,60,61,62]. At the cellular level, the presence of cyp1a protein has been observed in isolated immune cells of rainbow trout [52]. Together, these findings indicate that teleostean immune organs and cells possess the capacity for the biotransformation of xenobiotics such as PAHs, although cyp1a enzyme activities and biotransformation rates appear to be clearly lower compared to the liver [44,58,61]. 

In addition to *cyp1a* mRNA, we found transcripts of *ahr2α* and *ahr2β* in the immune organs and cells of rainbow trout. Our result on the presence of *ahr* transcripts in the head kidney is in line with previous studies reporting that the spleen, another immune organ of fish, expresses *ahr* transcripts: Yamauchi et al. [63] described this for the spleen of seabream (*Pagrus major*) and Lu et al. [64] for the spleen of goldfish (*Carassius auratus*). In both studies as well as in our study, *ahr* mRNA was detectable already in the immune organ of control fish, in the absence of exposure to AhR-activating xenobiotics, pointing to a substantial constitutional expression of the receptor in the immune system. 

RT-qPCR measurements on extracts of heterogenous organs such as the immune tissues do not reveal which cell type(s) within the organ display the *ahr* mRNA. Therefore, we employed FISH to test whether the *ahr* transcripts are indeed present in the immune cells. By this approach, we could demonstrate that the blood immune cells of rainbow trout express *ahr2* transcripts. This is the first direct localization of *ahr* transcripts in immune cells of a teleost fish species. Our result is corroborated by the study of Holen and Olsvik [65] who observed by means of Western blotting the presence of ahr protein in homogenates of head kidney leukocytes from cod (*Gadus morhua*). Together, the finding of AhR expression in immune cells of two fish species, trout and cod, suggest a more general presence of the receptor in the immune system of teleost fish.

The detection of the AhR in fish immune cells generates two questions: first, which immune cell types express the receptor, and second, is the receptor pathway functional? In mammals, the AhR pathway is restricted to specific immune cell populations such as B cells, T_H_17 cells, T_reg_ cells or dendritic cells [8,28]. The FISH data of our study clearly show that also in rainbow trout only some but not all immune cells are positive for *ahr* mRNA. In a previous study, we found that cyp1a protein is present in B cells and in granulocytes of rainbow trout [52]. Thus, these cells may also contain the *ahr* mRNA. Based on the morphological appearance of the *ahr*-positive cells in the FISH staining, the positive cells could be lymphocytes and neutrophils. Unpublished preliminary data from cell sorting experiments of our laboratory are supportive to this interpretation, as we found cell fractions enriched with B cells or ganulocytes to be positive for *ahr2* mRNA. Nevertheless, with the currently available data the question which immune cell types of rainbow trout express the AhR remains unresolved. Future studies should attempt to identify the nature of the AhR-positive immune cells of fish since this information is be crucial to understand both the physiological and the toxicological role of the receptor in the fish immune system. 

The second question is whether the AhR pathway is functional in the fish immune cells. The presence of *ahr2α* and *ahr2β* mRNA does not yet implicate functional signaling. The problem in studying this question is that to date it is not known which immune pathways and functions are regulated by the AhR in fish immune cells. Therefore, we decided to examine whether the expression of the *cyp1a* gene in the immune cells is regulated by a known AhR ligand, BaP. In all cells and tissues studied so far, the *cyp1a* gene was found to be tightly regulated by AhR (e.g. [17,60,66]). If the AhR pathway in the immune cells is functional, exposure to the AhR ligand, BaP, should result in *cyp1a* upregulation. Moreover, the presence of an AhR antagonist should inhibit this upregulation. The results of the present study show significant upregulation of *cyp1a* mRNA levels in the isolated immune cells of rainbow trout after exposure to the AhR ligand, BaP. To our knowledge, the only other study that has investigated the effects of xenobiotic exposure on CYP1A in fish immune cells is that of Holen and Olsvik [65]: these authors observed that exposure of isolated head kidney leukocytes of Atlantic cod to phenanthrene, another AhR-binding PAH, upregulated *cyp1a* transcript levels in the immune cells. The relative *cyp1a* induction was rather weak (factor of 2–3, compared to the factor 56 observed in the present study), what might be related to the fact that phenanthrene is a clearly less potent AhR agonist than BaP [67,68]. Independent of these differences in the magnitude of the induction response, the results of both Holen and Olsvik [65] and of our study show that AhR-activating xenobiotics are able to upregulate the transcription of the *cyp1a* gene in fish immune cells. That the upregulation of the *cyp1a* gene expression is indeed mediated through the AhR signaling pathway is indicated from the results of ANF experiment which showed that the presence of an AhR antagonist significantly reduces the *cyp1a* induction. Taken together, these findings provide the first evidence for the presence of functional AhR signaling in the immune cells of a teleost species. 

The next question that will have to be addressed is whether the AhR pathway in fish immune cells, beyond regulating biotransformation genes such a *cyp1a*, is also involved in the regulation of immune pathways and fucntions. Currently we have only indirect evidence for an immunoregulatory role of AhR in teleost immune cells. This indirect evidence comes from a few studies that investigated the response of immune genes in haematopoetic organs under exposure to AhR ligands. Hur et al. [41] reported that BaP exposure of olive flounder, *Paralichtyhs olivaceus,* altered the expression of a number of immune genes in the head kidney, including IL-1β, TNFα, IL-6, IL-8 and IFNγ. Holen and Olsvik [69] reported that treatment of isolated cod leukocytes with β-napthoflavone, another AhR ligand, interfered with the transcription of *cox2* and *il*-*8*, and with the secretion of leukotriene B_4_. In addition to altering gene expression and function of mature immune cells, the AhR pathway may also control the differentiation and proliferation of developing immune cells, as it is known to be the case in mammals [8,70]. In this context, the findings of Möller et al. [53] are of interest who observed that rainbow trout exposed to BaP experienced a significant decrease in the number of circulating T cells. This may be explained by an AhR-mediated impact of BaP on thymocyte differentiation and proliferation in the lymphoid organs. In this context, the finding of Spitsbergen et al. (1988) is of interest that exposure to TCDD and PCBs can cause lymphoid depletion in the fish thymus [71]. While all these data are still preliminary, they point to the possibility that the immunoregulatory function of the AhR is evolutionary conserved between mammals and fish. If the hypothesis of a physiological function of the AhR in the fish immune system could be substantiated in future studies, this would open an avenue to understand the mechanism(s) underlying the immunotoxic effects of AhR-binding contaminants in fish populations. 

## 4. Material and Methods

### 4.1. Fish Maintenance and Benzo[a]Pyrene (BaP) Exposure

All-female rainbow trout, *Oncorhynchus mykiss*, of 3 g body weight were obtained from DSM (Village Neuf, France) and reared in the coldwater fish facility of the Centre for Fish and Wildlife Health, University of Bern, Switzerland. The fishes were maintained at a water temperature of 15 ± 1 °C and were fed at 2% body weight with a commercial dry feed (Hokovit, Bützberg, Switzerland). Before sampling, the fishes were fasted for 24 h. The fishes were reared for 20 months until they had reached an average body weight of 1000 g. The experiments were performed in compliance with the Swiss legislation on animal experimentation under the permission number BE 44/15.

BaP (98% HPLC purity, 99.5 mol BaP/kg; Fluka, Buchs, Switzerland) was dissolved in 50 °C warm corn oil at a concentration of 25 mg/mL. For the administration of the BaP solution, the fishes were slightly anaesthesized with MS222 and then received a single intraperitoneal (i.p.) injection of 25 mg benzo[a]pyrene (BaP) per kg body weight. Control animals received corn oil only. Each injected trout was housed individually for five days in a 200 L glass tank supplied with flow-through water at 16.6 °C. The BaP treatment did not induce mortalities or any gross lesions. 

### 4.2. Sampling of Organs and Cells

The fishes were sacrificed by an overdose of MS222. For whole organ analyses, the liver and the head kidney were dissected and stored in RNA later (Ambion Inc, Austin, TX, USA) for RNA extraction. For the isolation of head kidney immune cells, the tissue was placed in L-15 medium containing 10 IU/mL heparin and then mechanically disaggregated by passing it through 250 µm and 125 µm nylon meshs. Peripheral blood cells were obtained by collecting blood from the caudal vein and diluting it with L-15 medium containing 10 IU/mL heparin. The cell suspensions we then layered onto a Ficoll separation solution (Biochrom AG, Berlin, Germany) and centrifuged at 400× *g*, 4 °C, for 40 min. The resulting immune cell fraction at the interphase was collected in L-15 medium, washed two times with PBS, and counted in a Neubauer chamber. The trypan blue exclusion assay was used to determine the number of viable cells. Viability in freshly isolated suspensions was <95%. Aliquots of ten million cells were stored in TRIzol reagent (Invitrogen, St. Louis, MO USA) for RNA extraction and kept at −80 °C until analysis.

### 4.3. In Vitro Exposure of Isolated Head Kidney Immune Cells

Isolated head kidney immune cells were plated in 24-well-plates (BD Falcon, Schaffhausen, Switzerland) at a density of 4 × 10^6^ cells per well. They were cultivated in an incubator (Biocenter 2001, Salvis, Rotkreuz, Switzerland) at 16 °C and 95% humidity. Cells were allowed to recover in L-15) with 2% Fetal calf Serum (FCS) for 24 h. Afterwards the attachment of the cells was controlled visually, and the medium was replaced by FCS-free L-15 medium. BaP and α-naphthoflavone (ANF) stock solutions were prepared in DMSO and added to the culture wells (final solvent concentration: 0.1%). Exposures were run over 48 hrs, with a medium change after 24 h. Solvent controls were included in each incubation. Each treatment was done in triplicates. Additional wells were used to test the cell viability at the end of the exposure period by mean of the resaruzin assay [67]. For sampling, cells were taken up in lysis buffer (peqLab RNAPure^TM^; peqlab, Erlangen, Germany) and stored frozen until RNA extraction. The in vitro experiments were conducted in at least three independent biological replicates, each of them using cells from different donor fishes. Within each incubation, all treatments were performed in three technical replicates. 

### 4.4. Fluorescence In Situ Hybridization (FISH)

The RNA probe was designed to detect both the AhR2α (accession No. AF065137) and the AhRβ, (accession No. AF065138α) isoforms of the receptor. For preparing the RNA-probe for AhR2, RNA was extracted from the liver of a rainbow trout using the RNAqueous^TM^ kit (Ambion) in accordance with the manufacturer’s protocol. The resulting RNA was reverse-transcribed to cDNA using the QIAGEN OneStep RT-PCR Kit (QIAGEN AG, Basel, Switzerland) and amplified into the AhR2 PCR-product for both AhR2α (accession No. AF065137) and Ahr2β (accession No. AF065138). The forward primer (5′-AATGGTCAGCCCTACAGGTG-3′) and the reverse primer (5′- CTGTTGTGGTCCGTT TGATG-3′) were synthesized by Microsynth AG (Balgach, Switzerland). The cDNA fragments (484bp) were separated by 1% agarose gel electrophoresis and cleaned up using a NucleoSpin Extract II (Macherey-Nagel, Düben, Germany). The purified cDNA was transformed using the pGEM^®^-T Easy Vector Systems (Promega, Madison, MI, USA) which contains T7 and SP6 promoters and DH5α competent cells. The plasmid DNA was purified by a QIAprep Spin Miniprep kit (QIAGEN AG, Hombrechtikon, Switzerland) and the insert was confirmed by ECORI enzyme reaction, followed by sequence analysis in an ABI PRISM (Applied Biosystems, Zug, Switzerland). The identification of the insert was performed by BLAST. The AhR2 plasmid DNA was linearized with *ndeI* and *ncoI* restriction enzymes and the linearized plasmid DNA was transcribed with either T7 or SP6 polymerase and a digoxigenin (DIG) RNA labeling kit (Roche Diagnostics AG, Rotkreuz, Switzerland) for anti-sense (positive probe) or sense (negative probe) DIG-RNA probe. The labeled RNA probes were diluted in nuclease-free water/deionized formamide (1/1, *v*/*v*) were stored at −20 °C before use. 

For performing FISH on the isolated immune cells from the peripheral blood, cell density was adjusted to 1 × 10^6^ cells/mL. Cells were fixed with Histochoice^TM^ (Electron Microscopy Sciences, Hatfield, PA, USA) for 10 min at 4 °C and adhered by cytospin centrifugation on a slide. Following repeated washes with PBS, the cells were treated with peroxidase at room temperature (RT) to block the endogenous peroxidase. The sections were acetylated with a solution containing 100 mM triethanolamine (pH 8.0) and 0.25% acetic anhydride for 10 min at RT, and rinsed twice in PBS for 5 min. For permeabilization, 0.2N HCl was used for 10 min at RT, after which the slides were washed 2× in PBS for 5 min. For pre-hybridization, the slides were treated with 4× saline sodium citrate (SSC) buffer for 5 min at RT, followed by a hybridization buffer consisting of 5 × SSC, 50% deionized formamide, 1× Denhardt’s, 12.5 mg/mL tRNA and 10% dextran sulfate for 60 min at 45 °C. For hybridization, the slides were incubated in a buffer containing either anti-sense or sense probe for 16 h at 45 °C. Next, slides were post-hybridized in 2× SSC and 0.1 × SSC, for at least 30 min and 45 min, respectively, at 50 °C. Slides were placed in TTBS buffer for 5 min at RT, and immersed in 0.5% blocking reagent (Roche Diagnostics) diluted in 100 mM Tris-HCl (pH 7.5) and 150 mM NaCl for 60 min at RT. For detection of digoxigenin (DIG), 1 µg/mL of a sheep anti DIG antibody (Roche Diagnostics) was applied on the cells for 60 min, followed by 74 µg/mL of a donkey anti sheep antibody (Jackson ImmunoResearch, West Grove, PA, USA) for 30 min, and 20 µg/mL of a Sheep PAP (Rockland, PA, USA) for 30 min at RT. Between these three incubation steps, slides were intensively washed in TTBS for the same incubation time. Finally, the signal was visualized by a tyramide signal amplification (TSA) fluorescence system (Perkin Elmer, Schwerzenbach, Switzerland). Nuclear staining was performed with 1 µg/mL of 4′,6-diamidino-2-phenylindole (DAPI, Roche Diagnostics). 

### 4.5. RNA Extraction and Quantitative Real-Time RT-PCR 

Samples in RNAlater or TRIzol, respectively, were homogenized and subjected to phase separation with bromochloropropane (Sigma-Aldrich, Buchs, Switzerland). The RNA was precipitated by isopropanol and ethanol washing and the resulting total RNA was dissolved in RNA-storage solution (Invitrogen). After the digestion of contaminating DNA using a TURBO- kit (Ambion), 1000 ng of RNA were reverse-transcribed to cDNA using a M-MLV reverse transcriptase system with random primer and dNTP as described in the manufacturer’s protocol (Promega). The TaqMan^®^ real-time RT-PCR was carried out in a 7500 Fast Real-time PCR System (Applied Biosystems). All samples were measured in triplicates with each sample of 12.5 µL total volume containing 1 µL of cDNA template, 0.5 µM of each forward and reverse primer, 0.2 µM of the probe and TaqMan^®^ Gene Expression Master Mix (Applied Biosystems). Primer and probe sequences for CYP1A (AF015660), AhR2α (AF065137) and AhR2β (AF065138) were as follows:

CYP1A
  Forward: 5′-TCCGGCACTCTTCCTTCCT-3′
  Reverse: 5′-GCCATTGAGGGATGTGTCCTT-3′
  Probe: 5′-CCGTTCATCATCCCACACTGCACG-3′
AhR2α
  Forward: 5′-TGTCAGATCCTCCCAATTTAAATG-3′
  Reverse: 5′-CTGAGGGAGACAAGAGATGAGTGA-3′
  Probe: 5′-CCCCTGACACTGAAGGCTCCCGT-3′
AhR2β
  Forward: 5′-GAATGTTATTTTGTTGGTGTTGTTGAAC-3′
  Reverse: 5′-GCATAGACTCCAGCGTTGTTACTC-3′
  Probe: 5′-TATAGCCGATTTACAGCAGAAGCGTCACCA-3′

The primers and probes of the target genes were synthesized by Microsynth (Balgach, Switzerland). Transcript levels were normalized against 18s RNA (primers and probes purchased from Applied Biosystems). 18s was selected as reference gene in preliminary experiments which compared the stability of 18s, *β-actin* and *ef1α*. 18s was measured using a commercial kit of Applied Biosystems with primers for conserved regions of eukaryotic 18s. Primer and probe efficiency of the target and reference genes were tested by generating dilution curves to calculate the amplification efficiency. The identity of the PCR products was confirmed by sequencing. In order to account for inter-plate variation, we distributed samples of the same treatment across plates, and we used a sample as reference which was measured i.e., each plate contained samples from the different treatments, in order to ensure comparability of data.

### 4.6. Statistical Analysis 

Expression of CYP1A and each AhR isoform was calculated by the 2(−ΔΔct) method and expressed means ±standard error [72]. The statistical difference between control and treatments was determined by ANOVA and two-sided Student’s t test. Differences with *p* < 0.05 were considered to be significant.

## 5. Conclusions

The results of this study provide evidence that rainbow trout immune organs and cells possess functional AhR signaling. This novel finding opens perspectives in comparative immunology as it suggests that the immunological role of the AhR is evolutionary conserved between fish and mammals. This is of relevance since fish species are increasingly used as model organisms in immunological research. Beyond, our finding opens perspectives in environmental toxicology as it points to xenobiotic interference with AhR signaling as mechanism underlying the well documented immunotoxic effects of AhR-binding PHAHs and PAHs in fish. What is currently missing to better understand the physiological and toxicological role of the AhR in the fish immune system is insight into which immune cell populations express the AhR pathway, and which specific immune pathways and function are regulated by the AhR. 

## Figures and Tables

**Table 1:** Induction of *cyp1a* mRNA transcripts in organs and cells of BaP-exposed rainbow trout relative to *cyp1a* transcript levels in the respective organs and cells of control rainbow trout.

Organ	Tissue/Cell	Control	BaP
L	Whole organ	1 ± 0.37	59 ±16 *
HK	Whole organ	1 ± 0.47	21 ± 1 *
HK	Total immune cells	1 ± 0.27	56 ± 16 *
PBL	Total immune cells	1 ± 0.60	92 ± 24 *

*cyp1a* gene expression levels in liver (L), head kidney (HK), immune cells isolated from head kidney and immune cells isolated from peripheral blood (PBL) of BaP-exposed rainbow trout relative to control fish. Data were analyzed using the 2^−(∆∆ct)^ method. Gene expression levels in organs and cells of BaP-exposed trout were compared to the mRNA levels in the respective organs and cells of the control trout, which were set to be 1. Data represent mean values ± SD from 10 fish per treatment. Asterisks indicate significant differences to control (*p* < 0.05). PBL = peripheral blood immune cells.

**Table 2:** Induction of *ahr2α* mRNA mRNA transcripts in organs and cells of BaP-exposed rainbow trout relative to *ahr2α* transcript levels in the respective organs and cells of control rainbow trout.

Organ	Tissue/Cell	Control	BaP
L	Whole organ	1 ± 0.36	1.73 ± 0.05 *
HK	Whole organ	1 ± 0.18	1.69 ± 0.35 *
HK	Total immune cells	1 ± 0.34	1.37 ± 0.53
PBL	Total immune cells	1 ± 0.28	1.09 ± 0.30

*ahr2α* gene expression levels in liver (L), head kidney (HK), immune cells isolated from head kidney and immune cells isolated from peripheral blood (PBL) of BaP-exposed rainbow trout relative to control fish. Data were analyzed using the 2^−(∆∆ct)^ method. Gene expression levels in organs and cells of BaP-exposed fish were compared to the mRNA levels in the respective organs and cells of the control fish, which were set to be 1. Data represent mean values ±SD from 10 fish per treatment. Asterisks indicate significant differences to control (*p* < 0.05). PBL = peripheral blood immune cells.

**Table 3:** Induction of *ahr2β* mRNA mRNA transcripts in organs and cells of BaP-exposed rainbow trout relative to *ahr2β* transcript levels in the respective organs and cells of control rainbow trout.

Organ	Cell Type	Control	BaP
L	Whole organ	1 ± 0.31	1.48 ± 0.33
HK	Whole organ	1 ± 0.17	1.32 ± 0.46
HK	Total immune cells	1 ± 0.43	1.36 ± 0.56
PBL	Total immune cells	1 ± 0.31	0.90 ± 0.27

ahr2α gene expression levels in liver (L), head kidney (HK), immune cells isolated from head kidney and immune cells isolated from peripheral blood (PBL) of BaP-exposed rainbow trout relative to control fish. Data were analyzed using the 2^−(∆∆ct)^ method. Gene expression levels in organs and cells of BaP-exposed fish were compared to the mRNA levels in the respective organs and cells of the control fish, which were set to be 1. Data represent mean values ± SD from 10 fish per treatment. Asterisks indicate significant differences to control (*p* < 0.05). PBL = peripheral blood immune cells.
